# Genome-Wide Association Study Identifies a Novel Susceptibility Locus at 12q23.1 for Lung Squamous Cell Carcinoma in Han Chinese

**DOI:** 10.1371/journal.pgen.1003190

**Published:** 2013-01-17

**Authors:** Jing Dong, Guangfu Jin, Chen Wu, Huan Guo, Baosen Zhou, Jiachun Lv, Daru Lu, Yongyong Shi, Yongqian Shu, Lin Xu, Minjie Chu, Cheng Wang, Ruyang Zhang, Juncheng Dai, Yue Jiang, Dianke Yu, Hongxia Ma, Xueying Zhao, Zhihua Yin, Lei Yang, Zhiqiang Li, Qifei Deng, Songyu Cao, Zhenzhen Qin, Jianhang Gong, Chongqi Sun, Jiucun Wang, Wei Wu, Guoquan Zhou, Hongyan Chen, Peng Guan, Yijiang Chen, Xiangyang Liu, Li Liu, Pin Xu, Baohui Han, Chunxue Bai, Yuxia Zhao, Haibo Zhang, Ying Yan, Jibin Liu, Christopher I. Amos, Feng Chen, Wen Tan, Li Jin, Tangchun Wu, Zhibin Hu, Dongxin Lin, Hongbing Shen

**Affiliations:** 1 Department of Epidemiology and Biostatistics and Ministry of Education (MOE) Key Lab for Modern Toxicology, School of Public Health, Nanjing Medical University, Nanjing, China; 2 Section of Clinical Epidemiology, Jiangsu Key Laboratory of Cancer Biomarkers, Prevention, and Treatment, Cancer Center, Nanjing Medical University, Nanjing, China; 3 State Key Laboratory of Reproductive Medicine, Nanjing Medical University, Nanjing, China; 4 State Key Laboratory of Molecular Oncology and Department of Etiology and Carcinogenesis, Cancer Institute and Hospital, Chinese Academy of Medical Sciences and Peking Union Medical College, Beijing, China; 5 Institute of Occupational Medicine and Ministry of Education Key Laboratory for Environment and Health, School of Public Health, Tongji Medical College, Huazhong University of Science and Technology, Wuhan, China; 6 Department of Epidemiology, School of Public Health, China Medical University, Shenyang, China; 7 The Institute for Chemical Carcinogenesis, State Key Laboratory of Respiratory Disease, Guangzhou Medical College, Guangzhou, China; 8 State Key Laboratory of Genetic Engineering, Center for Fudan–VARI Genetic Epidemiology and MOE Key Laboratory of Contemporary Anthropology, School of Life Sciences, Fudan University, Shanghai, China; 9 Bio-X Center and Affiliated Changning Mental Health Center, Ministry of Education Key Laboratory for the Genetics of Developmental and Neuropsychiatric Disorders, Shanghai JiaoTong University, Shanghai, China; 10 Department of Thoracic Surgery and Oncology, First Affiliated Hospital of Nanjing Medical University, Nanjing, China; 11 Department of Thoracic Surgery, Affiliated Cancer Hospital of Nanjing Medical University, Jiangsu Cancer Hospital, Nanjing, China; 12 Department of Thoracic Surgery, Cancer Hospital, Chinese Academy of Medical Sciences and Peking Union Medical College, Beijing, China; 13 Cancer Center of Union Hospital, Tongji Medical College, Huazhong University of Science and Technology, Wuhan, China; 14 Department of Oncology, Wuhan Iron and Steel Group/Corporation Staff-Worker Hospital, Wuhan, China; 15 Department of Respiratory Disease, Shanghai Chest Hospital, Shanghai Jiaotong University, Shanghai, China; 16 Department of Respiratory Disease, Zhongshan Hospital, Fudan University, Shanghai, China; 17 Department of Radiation Oncology, First Affiliated Hospital of China Medical University, Shenyang, China; 18 Department of Radiotherapy, Shenyang Northern Hospital, Shenyang, China; 19 Department of Surgery, Nantong Cancer Hospital, Nantong, China; 20 Department of Genetics, University of Texas M. D. Anderson Cancer Center, Houston, Texas, United States of America; University of Oxford, United Kingdom

## Abstract

Adenocarcinoma (AC) and squamous cell carcinoma (SqCC) are two major histological subtypes of lung cancer. Genome-wide association studies (GWAS) have made considerable advances in the understanding of lung cancer susceptibility. Obvious heterogeneity has been observed between different histological subtypes of lung cancer, but genetic determinants in specific to lung SqCC have not been systematically investigated. Here, we performed the GWAS analysis specifically for lung SqCC in 833 SqCC cases and 3,094 controls followed by a two-stage replication in additional 2,223 lung SqCC cases and 6,409 controls from Chinese populations. We found that rs12296850 in *SLC17A8*-*NR1H4* gene region at12q23.1 was significantly associated with risk of lung SqCC at genome-wide significance level [additive model: odds ratio (OR) = 0.78, 95% confidence interval (CI) = 0.72–0.84, *P* = 1.19×10^−10^]. Subjects carrying AG or GG genotype had a 26% (OR = 0.74, 95% CI = 0.67–0.81) or 32% (OR = 0.68, 95% CI = 0.56–0.83) decreased risk of lung SqCC, respectively, as compared with AA genotype. However, we did not observe significant association between rs12296850 and risk of lung AC in a total of 4,368 cases with lung AC and 9,486 controls (OR = 0.96, 95% CI = 0.90–1.02, *P* = 0.173). These results indicate that genetic variations on chromosome 12q23.1 may specifically contribute to lung SqCC susceptibility in Chinese population.

## Author Summary

Previous genome-wide association studies (GWAS) strongly suggested the importance of genetic susceptibility for lung cancer. However, the studies specific to different histological subtypes of lung cancer were limited. We performed the GWAS analysis specifically for lung squamous cell carcinoma (SqCC) with 570,009 autosomal SNPs in 833 SqCC cases and 3,094 controls and replicated in additional 2,223 lung SqCC cases and 6,409 controls from Chinese populations (822 SqCC cases and 2,243 controls for the first replication stage and 1,401 SqCC cases and 4,166 controls for the second replication stage). We found a novel association at rs12296850 (*SLC17A8*-*NR1H4*) on12q23.1. However, rs12296850 didn't show significant association with risk of lung adenocacinoma (AC) in 4,368 lung AC cases and 9,486 controls. These results indicate that genetic variations on chromosome 12q23.1 may specifically contribute to lung SqCC susceptibility in Chinese population.

## Introduction

Lung cancer is the most commonly diagnosed cancer and the leading cause of cancer death around the world [1]. Adenocarcinoma (AC) and squamous cell carcinoma (SqCC) are two major histological subtypes of lung cancer [2]. Although tobacco smoking increases the risk of all major histological subtypes of lung cancer, it appears to be stronger for SqCC than AC [3]. Different spectra and frequencies of “driver” mutations have been described between lung AC and SqCC and result in a histology-specific therapy [4]. These evidences support a histology-specific pathogenesis process and biological characteristics of lung cancer, and studies specifically focused on individual histological subtype are required for understanding lung carcinogenesis.

Several large genome-wide association studies (GWAS) of lung cancer have been conducted to uncover genetic factors associated with lung cancer risk [5]–[15] (Table S1). Three loci at 5p15, 6p21 and 15q25 were initially identified to contribute to the susceptibility to lung cancer in populations of European ancestry [5], [6], [16]–[18]. These findings have provided new clues for the mechanism of lung cancer development. Interestingly, some of these loci reflected different associations across lung cancer histology. For example, the 5p15 locus defined by rs2736100 showed stronger association with AC in populations of both European [7] and Asian [19] ancestries. However, most of lung cancer GWAS combined lung cancer cases with multiple subtypes of histology together when compared with controls in the discovery stage, making it difficult to identify histology-specific susceptibility loci due to dilution of effect.

With efforts to determine genetic variants associated with a specific type of lung cancer, two GWAS of lung AC have been conducted in populations of eastern Asian. Hsiung *et al.* performed a GWAS of AC and subsequent replications in never-smoking females and further confirmed that rs2736100 at 5p15 is associated with risk of lung AC [20]. Recently, Miki *et al.* carried out a GWAS of lung AC in Japanese and Korean populations and identified a new susceptibility locus at *TP63* on 3q28 [13], which have also been confirmed by following studies [11], [21]. Interestingly, Landi *et al.* conducted a lung cancer histology-specific association study in 917 selected genes with 19,802 SNPs in the HuGE-defined “inflammation” pathway using available GWAS data from populations of European descent, and identified a locus at 12p13.33 associated with SqCC risk [15]. These evidences suggest the importance of exploring susceptibility loci by subtypes in lung cancer.

Recently, we conducted a three-stage GWAS for overall lung cancer in the Han Chinese populations and identified two new loci at 13q12.12 and 22q12.2 that were consistently associated with multiple subtypes of lung cancer [11]. Here, in order to identify genetic variants across whole genome specifically related to lung SqCC risk, we carried out the GWAS analysis in 833 cases with lung SqCC and 3,094 controls (Nanjing study: 428 cases and 1,977 controls; and Beijing study: 405 cases and 1,117 controls), and further evaluated suggestive associations involving lung SqCC risk by a two-stage replication with a total of 2,223 cases with lung SqCC and 6,409 controls in the Han Chinese populations.

## Results

After filtering by standard quality-control procedures, a total of 3,927 subjects (833 lung SqCC cases and 3,094 controls) with 570,009 SNPs were qualified for further GWAS analysis (Table S2). A quantile-quantile plot using *P* values from additive model showed a relatively low inflation factor (λ = 1.04), suggesting a low possibility of false-positive associations due to population substructure (Figure S1). After excluding the SNPs at reported loci of our previous study [11], *P*-value on a -log scale for each SNP was plotted by location on chromosome (i.e., Manhattan plot; Figure S2).

We determined promising SNPs associated with risk of lung SqCC based on *P* value of ≤1×10^−4^ in additive model and consistent associations between Nanjing and Beijing studies (*P*<0.01 with the same direction of associations). After linkage disequilibrium (LD) analysis (excluding 9 SNPs at r^2^ of 0.8; Table S4), 14 autosome SNPs were selected to be further evaluated in the first replication stage (Replication I) including 822 cases with lung SqCC and 2,243 controls (Table S3). Three SNPs at 6p22.2 (rs16889835), 11p15.1 (rs7112278) and 12q23.1 (rs12296850) that were confirmed in the Replication I were further assessed in the second replication stage (Replication II) using additional 1,401 cases and 4,166 controls (Tables S5). In the Replication II, rs12296850 at 12q23.1 remained to be significantly associated with risk of lung SqCC (OR = 0.82, 95%CI = 0.74–0.91, *P* = 3.47×10^−4^), consistent with those observed in the GWAS stage (OR = 0.73, 95%CI = 0.63–0.86, *P* = 9.30×10^−5^) and the fist replication stage (OR = 0.75, 95%CI = 0.63–0.88, *P* = 5.08×10^−4^) (Table S5; Table 1). After combining results from the GWAS and two-stage replications, rs12296850 was associated with the risk of lung SqCC at genome-wide significance level (*P*<5.0×10^−8^), and the OR for additive model is 0.78 (95%CI = 0.72–0.84, *P*_combined_  = 1.19×10^−10^). The combined ORs for the heterozygote (AG) and minor homozygote (GG) are 0.74 (95% CI = 0.67–0.81) and 0.68 (95%CI = 0.56–0.83), respectively, as compared with major homozygote (AA) (Table 1).

**Table 1:** Summary of GWAS scan and replication studies for association between rs12296850 at 12q23.1 and risk of lung squamous cell carcinoma (SqCC).

Study	GG/AG/AA genotypes		G allele frequency		OR_het_	OR_homo_	OR_add_	*P*_add_ a
	Cases	Controls	Cases	Controls	(95% CI)^a^	(95% CI)^a^	(95% CI)^a^	
GWAS	35/287/511	185/1204/1705	0.214	0.254	0.76(0.63–0.92)	0.50(0.32–0.77)	0.73(0.63–0.86)	9.30×10^−5^
Replication I	38/254/517	127/856/1241	0.204	0.250	0.69(0.57–0.85)	0.68(0.43–1.06)	0.75(0.63–0.88)	5.08×10^−4^
Replication II	86/447/832	289/1547/2235	0.227	0.261	0.77(0.67–0.88)	0.77(0.59–1.01)	0.82(0.74–0.91)	3.47×10^−4^
Combined All	159/988/1860	601/3607/5181	0.217	0.256	0.74(0.67–0.81)	0.68(0.56–0.83)	0.78(0.72–0.84)	1.19×10^−10^

a Adjusted for age, gender, pack-year of smoking and the first principle component in GWAS, and for age, gender and pack-year of smoking in replication studies; OR_het_: AG *vs.* AA; OR_homo_: GG *vs.* AA; OR_add_ and *P_add_*: derived from additive model.

**Figure 1:**
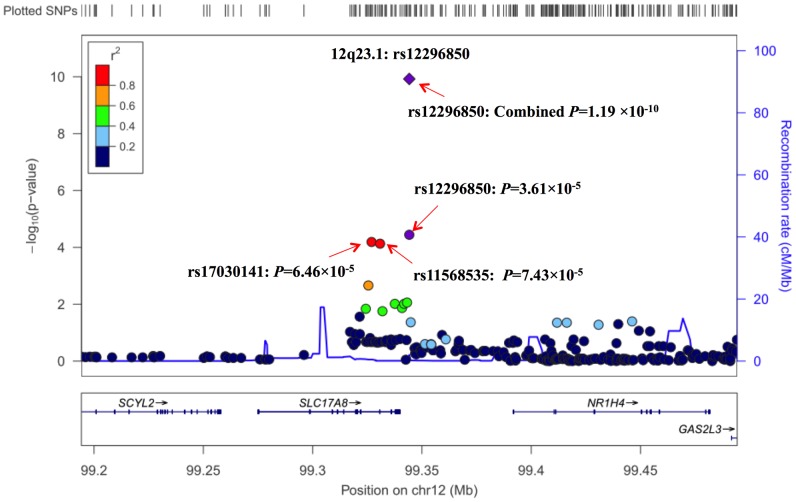
Regional plot of the identified marker rs12296850 at 12q23.1. Results (−log10 *P*) are shown for SNPs in the region flanking 150 kb on either side of rs12296850. The marker SNP are shown in purple and the *r* ^2^ values of the rest of the SNPs are indicated by different colors. The genes within the region of interest are annotated, with arrows indicating transcription direction.

To further characterize the association of genetic variants at 12q23.1 with lung SqCC risk, we performed imputation analyses based on CHB+JPT data of 1000 Genomes Project (released at June 2010). In a 300-kb region around rs12296850, 243 imputed SNPs at imputed r^2^>0.5 and MAF>0.05 were evaluated with association of lung SqCC risk. As shown in Figure 1 and Table S6, two SNPs, rs17030141 and rs11568535 having strong LD (r^2^>0.9) with rs12296850, showed similar associations with risk of lung SqCC at a *P* value of 6.46×10^−5^ and 7.43×10^−5^, respectively.

We further conducted stratification analysis on the association between rs12296850 at 12q23.1 and lung SqCC risk by age, gender and smoking dose. As shown in Table S7, none of different associations were significantly observed between subgroups. In addition, we did not detect significant interaction between rs12296850 and smoking on lung SqCC risk. Similar associations were observed among populations of Nanjing and Shanghai, Beijing, and Shenyang, and no significant heterogeneity between populations was detected for the association, though a non-significant association was shown in Guangzhou population (Figure S3).

To investigate whether the variant rs12296850 was SqCC-specific, we further evaluated the association between rs12296850 and the risk of lung AC and small cell carcinoma (SCC) using the shared controls as SqCC study for each stage. We found that rs12296850 was not consistently associated with risk of lung AC in the three stages (GWAS: OR = 0.85, 95%CI = 0.76–0.95; Replication I: OR = 1.08, 95%CI = 0.96–1.22; Replication II: OR = 0.96, 95%CI = 0.88–1.05) (Table 2). After combining three stages, rs12296850 was not significantly associated with lung AC risk (OR = 0.96, 95%CI = 0.90–1.02, *P*  = 0.173). Similarly, rs12296850 was not consistently associated with lung SCC risk with a combined OR of 0.89 (95%CI = 0.79–1.01; *P* = 0.073) (Table 2). These results indicate that rs12296850 at 12q23.1 may be a specific susceptibility locus to lung SqCC in Chinese population.

**Table 2:** Association between rs12296850 at 12q23.1 and risk of lung adenocarcinoma (AC) and small cell carcinoma (SCC).

Histology	Study	GG/AG/AA genotypes		G allele frequency		OR_het_	OR_homo_	OR_add_	*P*_add_ a
		Case	Control	Case	Control	(95% CI)^a^	(95% CI)^a^	(95% CI)^a^	
AC	GWAS	70/452/782	184/1200/1693	0.227	0.255	0.81(0.70–0.93)	0.80(0.60–1.08)	0.85(0.76–0.95)	3.47×10^−3^
	Replication I	101/403/636	127/856/1241	0.265	0.250	0.91(0.78–1.07)	1.56(1.17–2.08)	1.08(0.96–1.22)	0.200
	Replication II	139/667/1064	289/1547/2235	0.253	0.261	0.91(0.81–1.03)	1.01(0.81–1.26)	0.96(0.88–1.05)	0.393
	Combined All	310/1522/2482	600/3603/5169	0.248	0.256	0.88(0.81–0.95)	1.08(0.93–1.25)	0.96(0.90–1.02)	0.173
SCC	GWAS	6/60/112	184/1200/1693	0.202	0.255	0.71(0.51–0.99)	0.45(0.19–1.04)	0.69(0.53–0.92)	9.86×10^−3^
	Replication I	7/51/85	127/856/1241	0.227	0.250	0.87(0.60–1.25)	0.75(0.33–1.71)	0.87(0.64–1.17)	0.345
	Replication II	38/157/261	289/1547/2235	0.256	0.261	0.89(0.72–1.10)	1.11(0.77–1.62)	0.98(0.83–1.15)	0.792
	Combined All	51/268/458	600/3603/5169	0.238	0.256	0.83(0.70–0.97)	0.92(0.68–1.26)	0.89(0.79–1.01)	0.073

a Adjusted for age, gender and pack-year of smoking; OR_het_: AG *vs.* AA; OR_homo_: GG *vs.* AA; OR_add_ and *P_add_*: derived from additive model.

To characterize the functional relevance of the rs12296850, we further evaluated the relationship of this variant with the expression levels of two surrounding genes (*NRIH4* and *SLC17A8*). We examined *NRIH4* mRNA levels in 46 paired lung cancer tumor and adjacent non-tumor tissues using quantitative RT-PCR, and observed that the relative expression of *NRIH4* in adjacent non-tumor tissues was significantly higher in subjects with G allele of rs12296850 (n = 18) as compared with those carrying AA genotype (n = 28) (AG/GG: 0.54±0.25 versus AA: 0.36±0.19, *P* = 0.008)(Figure S4). Similar but non-significant results were also observed in tumor tissues (AG/GG: 0.50±0.22 versus AA: 0.39±0.26, *P* = 0.143). However, the mRNA expression level of *SLC17A8* could not be detectable (Ct>40) in all of the adjacent non-tumor tissues (n = 46) and most of tumor tissues (n = 43) whereas only 3 subjects were measured with low expression levels in tumor tissues (Ct = 33.7, 36.1 and 39.0).

## Discussion

In this study, we conducted a GWAS analysis in specific to lung SqCC in Chinese populations and identified a novel locus at 12q23.1 (lead SNP: rs12296850) that was specifically associated with lung SqCC. In our prior GWAS on overall lung cancer, we also showed genome-wide significant associations of loci at 3q28, 5p15.33, 13q12.12, and 22q12.2 with lung SqCC in stratification analysis [11]. Unlike previous study designed for overall lung cancer followed by a ‘post-hoc’ analysis on lung SqCC, the current study directly evaluated genetic variants across genome that might be specifically associated with lung SqCC risk. The identified locus was further assessed whether it was also associated with lung AC or SCC risk. This study represents an improved approach on exploring subtype-specific susceptibility loci for diseases with heterogeneous phenotypes, such as lung cancer.

We also evaluated the association of the SNP rs12296850 with SqCC risk in lung cancer GWAS data of European descent from MD Anderson Cancer Center (MDACC) [16]. After imputation based on HapMap 2 CEU population, rs12296850 was not significantly associated with SqCC risk (OR = 0.80, 95%CI: 0.52–1.24; *P* = 0.325) in 306 SqCC cases and 1,135 controls from the MDACC GWAS. The inconsistent results may be due to small sample size of MDACC study and different genetic backgrounds between Chinese and European descents. The minor allele (G) frequency of rs12296850 in Chinese population (>0.20 for all three stages) is more common than that in MDACC (0.053). The relative small sample size and low frequency may result in a negative result due to limited statistical power. In addition, the subjects of MDACC GWAS were all smokers, which may not represent the similar target population used in our study. However, at this stage, we have no substantial evidence to extend our findings to other populations, and further studies in other populations are required to further confirm our findings.

Genomic alterations on chromsome12q23 have been frequently linked to a spectrum of cancers, including non-small cell lung cancer (NSCLC), prostate cancer, adenoid cystic carcinoma and oligodendrogliomas and colorectal carcinoma [22]–[26]. For NSCLC, cigarette smoking dose has been associated with copy number alterations in12q23 [26]. In addition, chromosomal gains at 12q23–24.3 facilitated tumour progression and metastasis of lung SqCC and may serve as potential predictors for this disease [27]. These evidences as well as our findings collectively suggested the importance of chromosome 12q23 in the development of lung cancer, especially for SqCC.

At 12q23.1, the lead SNP rs12296850 is located in 4.2 kb downstream of *SLC17A8* (encoding vesicular glutamate transporter 3) and 47.6 kb upstream of *NR1H4* (encoding a ligand-activated transcription factor). Correlation analysis results indicate that this SNP may be associated with the expression of *NR1H4*, a gene known as nuclear farnesoid X receptor (FXR). FXR is a member of the nuclear receptor family of transcription factors and highly expressed in the entero-hepatic system where it transcriptionally regulates bile acid and lipid metabolism [28]. Bile acids are natural ligands for the *FXR*, and the bile acid-FXR interaction has been suggested to be involved in the pathophysiology of a number of inflammatory-associated cancers [29], [30]. Loss of FXR increased tumor progression via promoting Wnt signaling by infiltrating neutrophils and macrophages, and elevated the tumor necrosis factor α (TNFα) production *in vivo* [30]. Furthermore, FXR was involved in CYP regulation through mutual repression with NF-kappaB which indirectly regulates the transcription of CYP genes [31]. Further studies are required to elucidate the potential role of *NR1H4* on SqCC development.

*SLC17A8* (also known as Vesicular Glutamate Transporter Type 3, *VGLUT3*) is a member of the solute carrier (SLC) superfamily encoding multiple transmembrane transporters that may involve in the development and progression of a number of diseases, including cancers [32]. Genetic variants in the urea transporter (UT) gene *SLC14A* were reported to be significantly associated with susceptibility to urinary bladder cancer in a GWAS of European population, whereas *SLC5A8* may function as a tumor suppressor gene whose silencing by epigenetic changes may contribute to carcinogenesis and progression of pancreatic cancer [33], [34]. However, the expression levels of *SLC17A8* were very low in lung cancer tumor and adjacent non-tumor tissues. Whether this gene involves in SqCC development is still unclear to date.

In addition, *SCYL2* and *GAS2L3* were another two genes around the SNP rs12296850 in a relatively long distance. *SCYL2* (also known as *CVAK104*) is located at 86.2 kb upstream of rs12296850, encoding a coated vesicle-associated kinase of 104 kDa. *SCYL2* can regulate the levels of frizzled 5 (Fzd5) via inducing lysosomal degradation, which probably inhibit the Wnt signaling pathway [35]. *GAS2L3*, encoding proteins with putative actins and microtubule binding domains, is located at 147.4 kb downstream of rs12296850. *GAS2L3* was reported to localize to the spindle midzone and the midbody during anaphase and cytokinesis, respectively, and to act as a novel target of DREAM and play an important role in accurate cell division [36]. However, expression quantitative trait loci (eQTL) analysis did not reveal any significant correlation between rs12296850 and the expressions of these two genes.

In this GWAS of lung SqCC in Chinese, we reported evidence that common genetic variants at 12q23.1 are implicated in the development of lung SqCC. Our findings highlight the importance of studying subtype of lung cancer and may provide new insight into the mechanism of SqCC. Further studies, such as resequencing this region followed by fine-mapping study and eQTL analysis in lung tissues as well as biochemical assays, may affiliate to determine causal variants at 12q23.1 that directly regulate the development of lung SqCC. In addition, the moderate sample size in GWAS scan stage may have decreased statistical power in the current study, and further studies with larger sample size or pooling multiple studies may promise to identify more SqCC-specific loci.

## Materials and Methods

### Study populations

A three-stage case-control study was designed to evaluate the associations between genetic variants across human genome and the risk of lung SqCC. Study subjects for GWAS scan of lung cancer and two-stage replication have been described elsewhere [11]. Briefly, the cases newly diagnosed with lung cancer were recruited from hospitals. The histology for each case was histopathologically or cytologically confirmed by at least two local pathologists. Cancer-free control subjects were recruited in local hospitals for individuals receiving routine physical examinations or in the communities for those participating screening of noncommunicable diseases. The controls were frequency-matched to lung cancer cases for age, gender and geographic regions. Demographic information was collected using standard questionnaire through interviews. Individuals were defined as smokers if they had smoked at an average of one cigarette or more per day and for at least one year in their lifetime; otherwise, subjects were considered as non-smokers. Smokers were considered as former smokers who quit for at least one year before recruitment. Both current and former smokers were divided into light and heavy smokers according to the threshold of 25 pack-year (median value among the controls).

The patients with lung SqCC and all of the controls that were included in previous GWAS of overall lung cancer [11] were considered as the cases and controls in the current study. As a result, 833 SqCC cases and 3,094 controls were included in the GWAS scan stage, including 428 cases and 1977 controls from Nanjing and Shanghai (Nanjing Study), and 405 cases and 1,117 controls from Beijing (Beijing Study). The first replication stage (Replication I) included 822 SqCC cases and 2,243 controls that were from Nanjing and Shanghai (235 cases and 754 controls) and Beijing (587 cases and 1,489 controls). The second replication stage (Replication II) included 1,401 SqCC cases and 4,166 controls that were from Nanjing and Shanghai (238 cases and 1,069 controls), Beijing (362 cases and 936 controls), Shenyang (306 cases and 1,027 controls) and Guangzhou (495 cases and 1,134 controls).

### Ethics statement

All study subjects provided informed consent and each study was approved by its respective institution's IRB.

### Quality control (QC) in GWAS

A total of 906,703 SNPs were genotyped in the GWAS scan in 844 lung SqCC cases and 3,160 controls by using Affymetrix Genome-Wide Human SNP Array 6.0 chips as described previously [11]. A systematic quality control (QC) procedure was applied to both SNPs and samples before association analysis. SNPs were excluded if they (i) did not map on autosomal chromosomes; (ii) had a call rate <95%; (iii) had a minor allele frequency (MAF) <0.05; or (iv) deviated from Hardy-Weinberg equilibrium (*P*<1×10^−5^ in all GWAS samples or *P*<1×10^−4^ in either of the Nanjing Study or the Beijing Study samples). We removed samples with low genotype call rates <0.95 (3 subjects) and ambiguous gender (4 subjects). Unexpected duplicates or probable relatives (52 subjects) identified by pairwise identity-by-state comparisons were also excluded according to their PI_HAT value in PLINK (all PI_HAT>0.25). Heterozygosity rates were calculated, and samples were excluded if they were more than 6 s.d. away from the mean (12 subjects were excluded). We detected population outliers using a method based on principle component analysis and 6 subjects were removed. As a result, 833 lung SqCC cases and 3,094 controls with 570,009 SNPs remained after QC.

### SNP selection and genotyping in the replication study

After genome-wide association analyses, we selected SNPs for the first stage replication based on the following criteria: (i) SNPs had *P*≤1.0×10^−4^ for all GWAS samples; (ii) they showed consistent associations between the Nanjing study and the Beijing study at *P*≤1.0×10^−2^; (iii) they are not located in the same chromosome regions or genes of SNPs reported in previous GWAS; (iv) they had clear genotyping clusters; (v) only the SNP with the lowest *P* value was selected when multiple SNPs were observed in a strong linkage disequilibrium (LD) (r^2^≥0.8). As s results, a total of 23 SNPs satisfied the criteria (i), (ii), (iii) and (iv), and 14 SNPs survived according to criterion (v). Therefore, we genotyped these 14 SNPs in the first replication stage (Table S3) and the other 9 SNPs that were in strong LD with 14 selected SNPs were excluded from further analysis (Table S4). The SNPs showed significant associations with lung SqCC risk with *P*<0.05 in the first stage replication were selected for the second replication stage.

Genotyping were performed by using the TaqMan OpenArray Genotyping Platform (Applied Biosystems, Inc.) and the iPLEX Sequenom MassARRAY platform (Sequenom, Inc) for SNPs selected in the first replication stage, and TaqMan allelic discrimination Assay (Applied Biosystems, Inc.) for SNPs selected in the second replication stage. A series of methods was used to control the quality of genotyping: (i) case and control samples were mixed on each plate and genotyped without knowing the case or control status; (ii) two water controls in each plate were used as blank controls; (iii) five percent of the samples were randomly selected to repeat the genotyping, as blind duplicates, and the reproducibility was 100%; (iv) 1,347 samples were randomly selected and detected using both TaqMan Openarray platform and TaqMan assay for rs12296850, yielding a concordance rate of 99.97%.

### Statistical analysis

The statistical analysis methodology of our lung cancer GWAS was described previously [11]. In brief, genome-wide association analysis was performed using logistic regression analysis in additive model as implemented in PLINK 1.07 (see URLs). EIGENSTRAT 3.0 was used for the principal component analysis of population structure. Minimac software (see URLs) was used to impute untyped SNPs using the CHB+JPT data from the hg18/1000 Genomes database (released at June 2010) as reference set. Regional plot was generated using the LocusZoom 1.1(see URLs). R software (version 2.11.1; The R Foundation for Statistical Computing) was also used for statistical analysis and generating plots, including Q-Q plot and Manhattan plot.

### Tissue samples

To determine the expression levels of *NRIH4* and *SLC17A8*, we collected 46 paired lung cancer tissues from the patients who had undergone resection between June 2009 and April 2010 from the Nantong Cancer Hospital. All cases were histopathologically diagnosed lung cancer without radiotherapy or chemotherapy before surgical operation.

### Quantitative reverse transcription polymerase chain reaction

Quantitative reverse transcription polymerase chain reaction (qRT-PCR) was performed to determine the mRNA expressions of *NRIH4* and *SLC*17A8. RNAs from lung cancer tumor and adjacent non-tumor tissues were isolated with the Trizol reagent (Invitrogen). We used TaqMan gene expression probes (Applied Biosystems Inc.) to perform qRT-PCR assay. All real-time PCR reactions, including no-template controls and real-time minus controls, were run by using the ABI7900 Real-Time PCR System (Applied Biosystems Inc.) and performed in triplicate. β-actin gene was used to normalize the expression levels. A relative expression was calculated using the equation 2^−ΔCt^ (Ct, Cycle Threshold), in which ΔCt  = Ct _gene_−Ct _β-actin_.

### *Cis*-eQTL analysis

We applied the publicly available data from GTEx (Genotype-Tissue Expression) eQTL Browser, eQTL.Chicago.edu and Gene Expression Analysis Based on Imputed Genotypes (see URLs) to perform *cis*-eQTL analysis and evaluated the *cis* association between rs12296850 and the expression of nearby genes in a variety of cells/tissues, including lymphoblastoid cell lines [37]–[42], monocytes [43], fibroblasts [42], liver [44] and brain tissues [45].

### URLs

PLINK1.07, http://pngu.mgh.harvard.edu/~purcell/plink/; R 2.11.1 statistical environment, http://www.cran.r-project.org/; Minimac, http://genome.sph.umich.edu/wiki/Minimac ;LocusZoom 1.1, http://csg.sph.umich.edu/locuszoom/; GTEx (Genotype-Tissue Expression) eQTL Browser, http://www.ncbi.nlm.nih.gov/gtex/test/GTEX2/gtex.cgi ; eQTL.Chicago.edu, http://eqtl.uchicago.edu/cgi-bin/gbrowse/eqtl/; Gene Expression Analysis Based on Imputed Genotypes, http://www.sph.umich.edu/csg/liang/imputation/.

## Supporting Information

Figure S1 Quantile–Quantile plot of *P*-values in −log10 scale.
(TIF)

Figure S2 Genome-wide association results on lung SqCC in Han Chinese. Scatter plot of *P* values in −log10-scale from the additive model on 569,669 SNPs (833 cases and 3,094 controls). Note: SNPs located in the same chromosome regions or genes of SNPs reported in our previous GWAS were not included in the plot. Blue line: *P* = 1.0×10^−4^.
(TIF)

Figure S3 Association results for rs12296850 and lung SqCC risk by site of subjects collection.
(TIF)

Figure S4 The relative expression levels of *NRIH4* by rs12296850 genotypes in 46 paired lung cancer tumor and adjacent non-tumor tissues as measured by quantitative RT-PCR. Lines indicate the median with quartiles.
(TIF)

Table S1 Effects of GWAS identified loci for lung cancer on subtypes of lung cancer by histology.
(DOC)

Table S2 Summary description of the samples used in this study.
(DOC)

Table S3 Summary of associations between 14 SNPs and lung SqCC risk in GWAS selected for replication.
(DOC)

Table S4 SNPs satisfy the selection criteria for replication but are in strong linkage disequilibrium (r^2^>0.8) with selected SNPs.
(DOC)

Table S5 Summary of associations of 14 SNPs with risk of lung SqCC in GWAS scan and replication studies.
(DOC)

Table S6 SNPs at 12q13.1 associated with risk of lung SqCC at *P*<1.0×10^−4^.
(DOC)

Table S7 Stratification analysis on association of rs12296850 at 12q13.1 and lung SqCC risk.
(DOC)
