# Supplementary figures and images for: Genome-Wide Association Study Identifies a Novel Susceptibility Locus at 12q23.1 for Lung Squamous Cell Carcinoma in Han Chinese
Source: PLoS Genet. 2013 Jan 17;9(1):e1003190. doi: 10.1371/journal.pgen.1003190 (PMC3547794; doi:10.1371/journal.pgen.1003190)

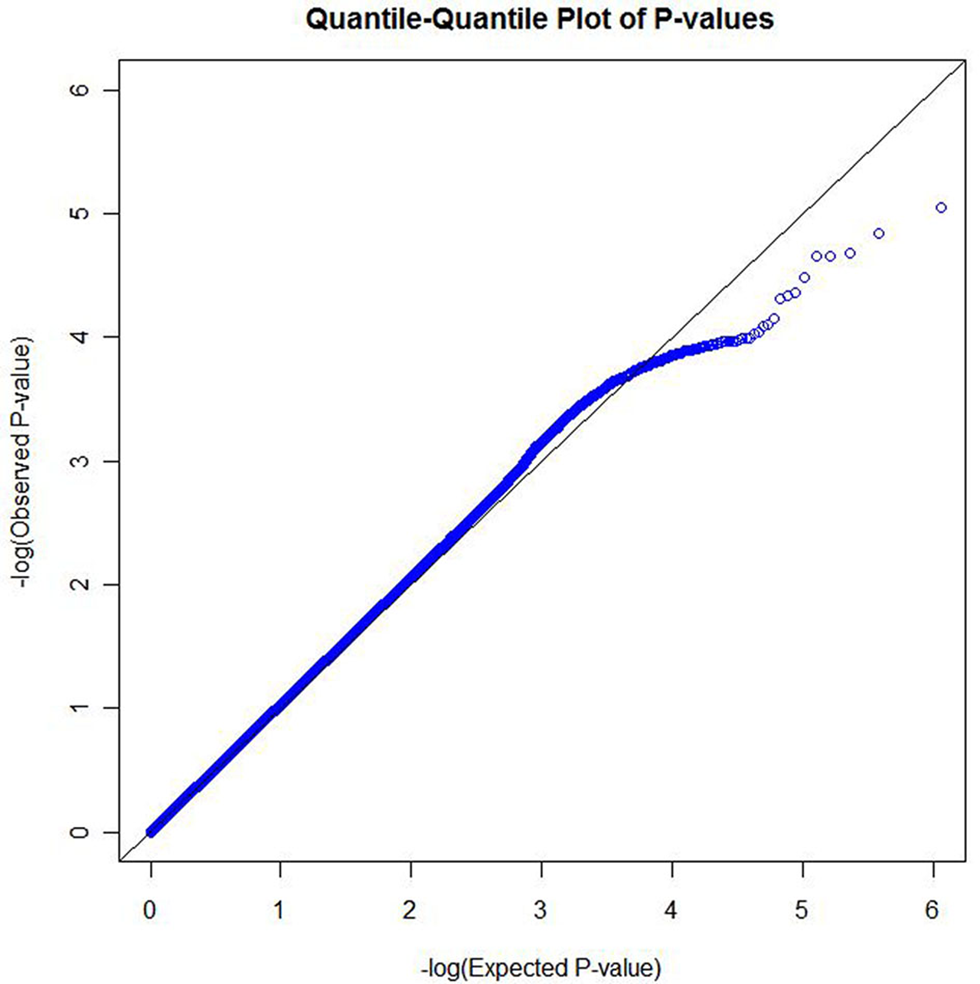

Supplement: Figure S1 — Quantile–Quantile plot of P-values in −log10 scale. (TIF) [file pgen.1003190.s001.tif]

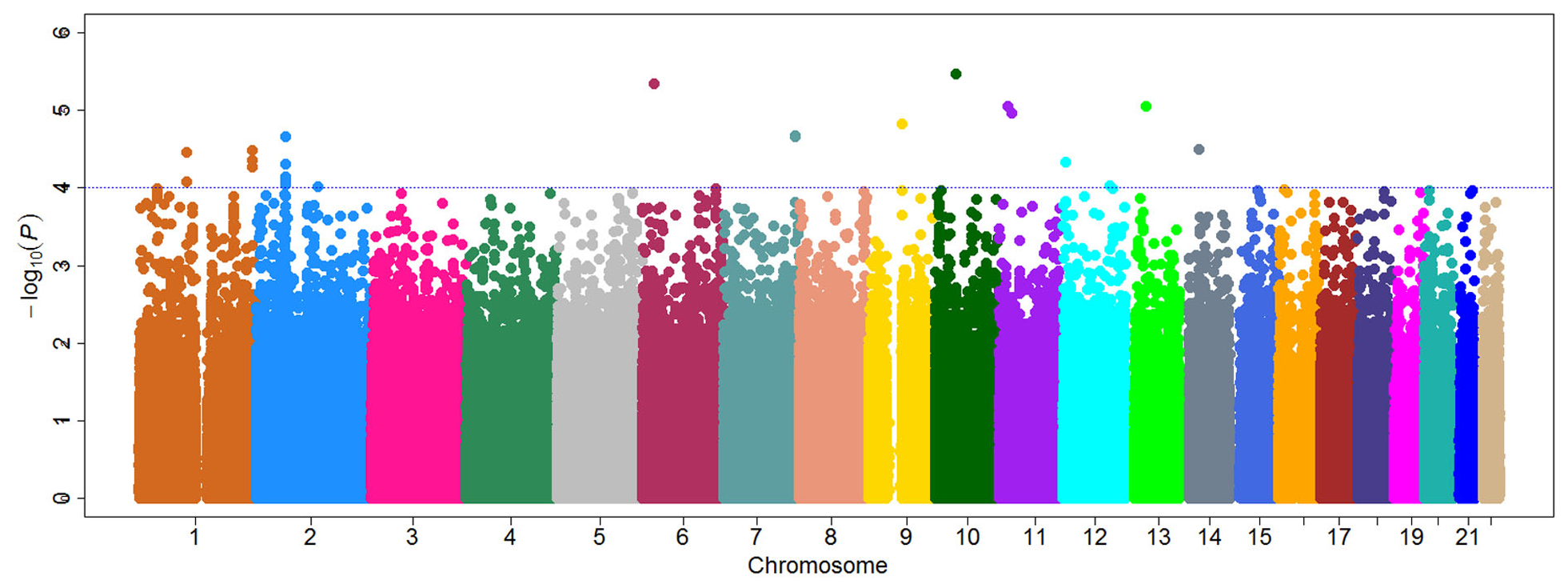

Supplement: Figure S2 — Genome-wide association results on lung SqCC in Han Chinese. Scatter plot of P values in −log10-scale from the additive model on 569,669 SNPs (833 cases and 3,094 controls). Note: SNPs located in the same chromosome regions or genes of SNPs reported in our previous GWAS were not included in the plot. Blue line: P = 1.0×10^−4^. (TIF) [file pgen.1003190.s002.tif]

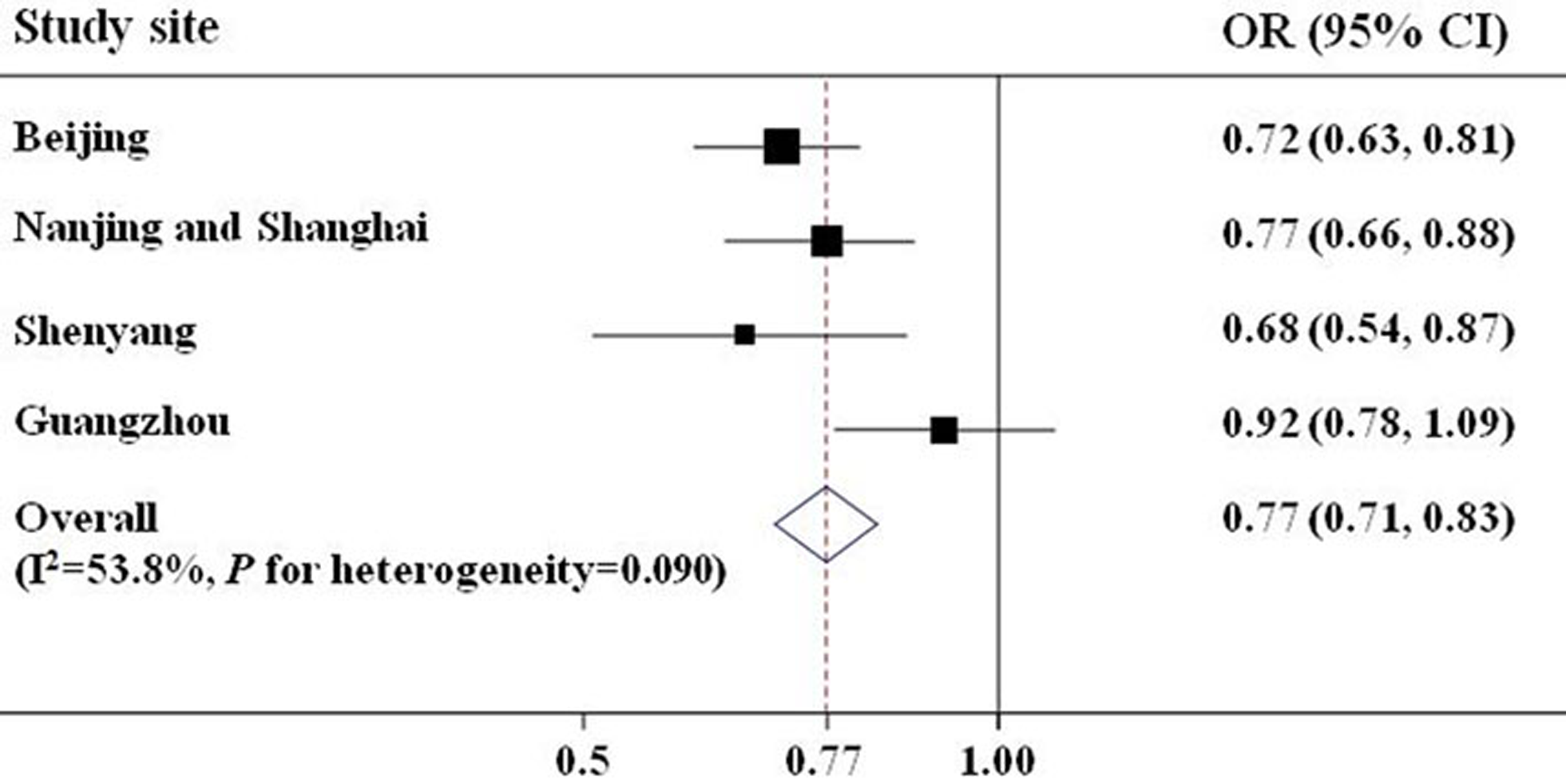

Supplement: Figure S3 — Association results for rs12296850 and lung SqCC risk by site of subjects collection. (TIF) [file pgen.1003190.s003.tif]

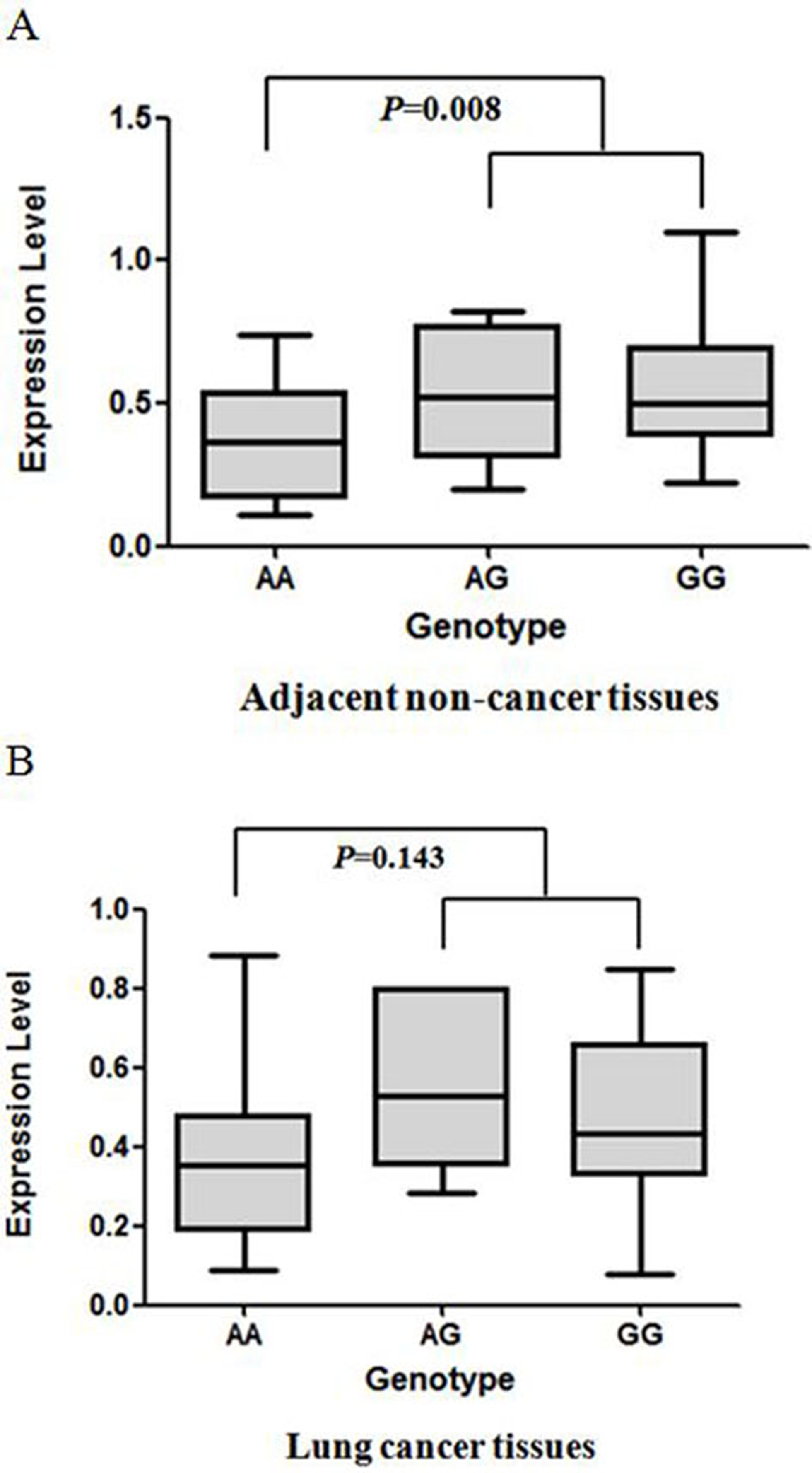

Supplement: Figure S4 — The relative expression levels of NRIH4 by rs12296850 genotypes in 46 paired lung cancer tumor and adjacent non-tumor tissues as measured by quantitative RT-PCR. Lines indicate the median with quartiles. (TIF) [file pgen.1003190.s004.tif]
