# Supplementary material for: Genome-Wide Association Study Identifies a Novel Susceptibility Locus at 12q23.1 for Lung Squamous Cell Carcinoma in Han Chinese
Source: PLoS Genet. 2013 Jan 17;9(1):e1003190. doi: 10.1371/journal.pgen.1003190 (PMC3547794; doi:10.1371/journal.pgen.1003190)
Supplement: Table S1 — Effects of GWAS identified loci for lung cancer on subtypes of lung cancer by histology. (DOC) [file pgen.1003190.s005.doc]

**Table S1.** Effects of GWAS identified loci for lung cancer on subtypes of lung cancer by histology

| SNP | Chr. | Alleles | AC^*^ | |  | SqCC^*^ | |  | SCC^*^ | | Ref. No. |
| --- | --- | --- | --- | --- | --- | --- | --- | --- | --- | --- | --- |
|  |  |  | OR | *P* |  | OR | *P* |  | OR | *P* |  |
| rs8034191 | 15q25.1 | T/C | 1.37 | 2×10^-10^ |  | 1.23 | 6×10^-6^ |  | 1.28 | 2×10^-4^ | 5 |
| rs4324798 | 6p22.1 | G/A | 1.17 | 5.90×10^-2^ |  | 1.51 | 3×10^-8^ |  | 1.33 | 6.00×10^-3^ | 5 |
| rs402710 | 5p15.33 | T/C | 1.19 | - |  | 1.21 | - |  | 1.14 | - | 6 |
| rs2736100 | 5p15.33 | T/G | 1.17 | - |  | 1.09 | - |  | 1.14 | - | 6 |
| rs31489 | 5p15.33 | C/A | 0.92 | 5.00×10^-3^ |  | 0.87 | 1.39×10^-5^ |  | 0.91 | 2.40×10^-2^ | 7 |
| rs12914385 | 15q25.1 | G/A | 1.31 | 3.32×10^-18^ |  | 1.30 | 8.72×10^-13^ |  | 1.37 | 2.31×10^-9^ | 7 |
| rs1051730 | 15q25.1 | G/A | 1.32 | 7.10×10^-19^ |  | 1.28 | 1.01×10^-10^ |  | 1.37 | 3.18×10^-9^ | 7 |
| rs2172706 | 1q21.3 | G/A | 0.75 | 3.10×10^-2^ |  | - | - |  | - | - | 8 |
| rs1470037 | 2p12 | A/C | 0.68 | 9.70×10^-3^ |  | - | - |  | - | - | 8 |
| rs1877116 | 4q13.1 | A/G | 0.74 | 2.73×10^-2^ |  | - | - |  | - | - | 8 |
| rs4897493 | 6q23.2 | T/C | 0.70 | 2.03×10^-2^ |  | - | - |  | - | - | 8 |
| rs2515373 | 11q22.1 | A/G | 0.73 | 6.22×10^-2^ |  | - | - |  | - | - | 8 |
| rs6488007 | 12p11.21 | A/G | 0.72 | 1.92×10^-2^ |  | - | - |  | - | - | 8 |
| rs16918924 | 12p11.21 | A/C | 0.63 | 4.00×10^-3^ |  | - | - |  | - | - | 8 |
| rs5945306 | Xq28 | T/C | 0.62 | 8.60×10^-3^ |  | - | - |  | - | - | 8 |
| rs2131877 | 3q29 | A/G | 1.27 | 9.90×10^-6^ |  | 1.38 | 4.18×10^-4^ |  | - | - | 9 |
| rs2352028 | 13q31.3 | G/A | 1.39 | 3.0×10^-4^ |  | - | - |  | - | - | 10 |
| rs4488809 | 3q28 | T/C | 1.32 | 9.62×10-^25^ |  | 1.24 | 1.28×10^-10^ |  | 1.05 | 3.98×10^-1^ | 11 |
| rs465498 | 5p15.33 | A/G | 0.76 | 8.02×10^-14^ |  | 0.72 | 3.65×10^-12^ |  | 0.8 | 3.28×10^-3^ | 11 |
| rs753955 | 13q12.12 | A/G | 1.2 | 6.41×10^-10^ |  | 1.19 | 6.70×10^-7^ |  | 1.12 | 6.15×10^-2^ | 11 |
| rs17728461 | 22q12.2 | C/G | 1.17 | 2.14×10^-6^ |  | 1.24 | 4.76×10^-8^ |  | 1.24 | 1.00×10^-3^ | 11 |
| rs36600 | 22q12.2 | G/A | 1.23 | 6.36×10^-7^ |  | 1.37 | 6.83×10^-10^ |  | 1.45 | 4.65×10^-6^ | 11 |
| rs11080466 | 18p11.22 | T/C | 0.66 | 1.19×10^-6^ |  | - | - |  | - | - | 12 |
| rs11663246 | 18p11.22 | C/T | 0.67 | 2.4×10^-6^ |  | - | - |  | - | - | 12 |
| rs10937405 | 3q28 | C/T | 1.31 | 7.26×10^-12^ |  | - | - |  | - | - | 13 |
| rs2853677 | 5p15.33 | T/C | 1.41 | 2.8×10^-40^ |  | - | - |  | - | - | 14 |
| rs7216064 | 17q24.3 | A/G | 1.2 | 7.4×10^-11^ |  | - | - |  | - | - | 14 |
| rs3817963 | 6p21.3 | A/G | 1.18 | 2.7×10^-10^ |  | - | - |  | - | - | 14 |
| rs6489769 | 12p13.33 | A/G | 1.2 | 2.3×10^-8^ |  | - | - |  | - | - | 15 |

^*^ ORs and *P* values were derived from corresponding studies for lung adenocarcinoma (AC), squamous cell carcinoma (SqCC), and small cell carcinoma (SCC).
