# Supplementary material for: Genome-Wide Association Study Identifies a Novel Susceptibility Locus at 12q23.1 for Lung Squamous Cell Carcinoma in Han Chinese
Source: PLoS Genet. 2013 Jan 17;9(1):e1003190. doi: 10.1371/journal.pgen.1003190 (PMC3547794; doi:10.1371/journal.pgen.1003190)
Supplement: Table S2 — Summary description of the samples used in this study. (DOC) [file pgen.1003190.s006.doc]

**Table S2.** Summary description of the samples used in this study

| **Variables** | **GWAS scan** | | | |  | **Replication I** | |  | **Replication II** | |
| --- | --- | --- | --- | --- | --- | --- | --- | --- | --- | --- |
|  | **Nanjing Study** | | **Beijing Study** | |  |  |  |  |  |  |
|  | **Case** | **Control** | **Case** | **Control** |  | **Case** | **Control** |  | **Case** | **Control** |
|  | **(n=428)** | **(n=1,977)** | **(n=405)** | **(n=1,117)** |  | **(n=822)** | **(n=2,243)** |  | **(n=1401)** | **(n=4,166)** |
| Age (Mean ± S.D.) | 62.29±9.24 | 59.39±9.77 | 60.63±10.05 | 62.29±9.25 |  | 60.06±9.04 | 56.51±12.61 |  | 60.53±10.61 | 58.78±11.41 |
| Gender |  |  |  |  |  |  |  |  |  |  |
| Male | 400 | 1225 | 378 | 873 |  | 745 | 1525 |  | 1130 | 2651 |
| Female | 28 | 752 | 27 | 244 |  | 77 | 718 |  | 271 | 1515 |
| Smoking Status |  |  |  |  |  |  |  |  |  |  |
| Current Smokers | 304 | 648 | 321 | 443 |  | 577 | 800 |  | 709 | 1384 |
| Former smokers | 81 | 91 | 43 | 142 |  | 140 | 34 |  | 275 | 330 |
| Never smokers | 43 | 1238 | 41 | 532 |  | 105 | 1409 |  | 417 | 2452 |
| Smoking Levels (Mean ± S.D.) | 45.94±27.53 | 31.01±20.05 | 45.64±31.37 | 32.47±19.93 |  | 43.39±26.10 | 26.20±18.70 |  | 39.06±25.79 | 24.53±18.18 |
| ≤ 25(Pack-years) | 80 | 331 | 100 | 231 |  | 181 | 455 |  | 325 | 1061 |
| > 25(Pack-years) | 305 | 408 | 264 | 354 |  | 536 | 379 |  | 659 | 653 |
