# Supplementary material for: Genome-Wide Association Study Identifies a Novel Susceptibility Locus at 12q23.1 for Lung Squamous Cell Carcinoma in Han Chinese
Source: PLoS Genet. 2013 Jan 17;9(1):e1003190. doi: 10.1371/journal.pgen.1003190 (PMC3547794; doi:10.1371/journal.pgen.1003190)
Supplement: Table S3 — Summary of associations between 14 SNPs and lung SqCC risk in GWAS selected for replication. (DOC) [file pgen.1003190.s007.doc]

**Table S3.** Summary of associations between 14 SNPs and lung SqCC risk in GWAS selected for replication

| **Chr.** | **SNP** | **Associated Gene** | **Major/Minor**  **Allele** | **MAF ^a^** | | **OR_all ^b^** | ***P*_all ^b^** | **OR_nj ^b^** | ***P*_nj ^b^** | **OR_bj ^b^** | ***P*_bj ^b^** |
| --- | --- | --- | --- | --- | --- | --- | --- | --- | --- | --- | --- |
|  |  |  |  | **Case** | **Control** |  |  |  |  |  |  |
| 1p21.2 | rs1445227 | RPSA | A/G | 0.195 | 0.164 | 1.39 | 3.50×10^-5^ | 1.45 | 7.57×10^-4^ | 1.34 | 1.00×10^-2^ |
| 1q43 | rs5009401 | MAP1LC3C | A/G | 0.249 | 0.287 | 0.73 | 3.26×10^-5^ | 0.74 | 3.17×10^-3^ | 0.74 | 9.01×10^-3^ |
| 2p15 | rs2167566 | USP34 | T/G | 0.337 | 0.382 | 0.75 | 4.90×10^-5^ | 0.74 | 1.07×10^-3^ | 0.74 | 1.96×10^-3^ |
| 2q21.1 | rs6724110 | LOC389033 | T/C | 0.399 | 0.455 | 0.78 | 9.49×10^-5^ | 0.79 | 1.00×10^-2^ | 0.78 | 6.95×10^-3^ |
| 6p22.2 | rs16889835 | tcag7.873 | C/T | 0.240 | 0.298 | 0.72 | 4.49×10^-6^ | 0.76 | 6.08×10^-3^ | 0.69 | 4.48×10^-4^ |
| 7q36.1 | rs10952289 | ABP1 | C/T | 0.169 | 0.126 | 1.47 | 2.23×10^-5^ | 1.48 | 1.12×10^-3^ | 1.52 | 5.89×10^-4^ |
| 9q21.11 | rs2039625 | PIP5K1B | T/A | 0.218 | 0.257 | 0.71 | 1.46×10^-5^ | 0.77 | 9.91×10^-3^ | 0.65 | 3.79×10^-4^ |
| 10q11.21 | rs10900189 | OR13A1 | C/T | 0.240 | 0.288 | 0.72 | 3.45×10^-6^ | 0.73 | 1.55×10^-3^ | 0.74 | 8.56×10^-3^ |
| 11p14.1 | rs10835161 | CCDC34 | A/C | 0.418 | 0.476 | 0.76 | 1.06×10^-5^ | 0.79 | 1.00×10^-2^ | 0.72 | 3.59×10^-4^ |
| 11p15.1 | rs7112278 | FAM10A4 | T/C | 0.195 | 0.254 | 0.71 | 8.84×10^-6^ | 0.73 | 3.34×10^-3^ | 0.69 | 6.88×10^-4^ |
| 12p13.31 | rs216905 | VWF | C/T | 0.135 | 0.093 | 1.52 | 4.61×10^-5^ | 1.67 | 1.12×10^-4^ | 1.54 | 2.59×10^-3^ |
| 12q23.1 | rs12296850 | SLC17A8-NR1H4 | A/G | 0.214 | 0.254 | 0.73 | 9.30×10^-5^ | 0.70 | 7.37×10^-4^ | 0.73 | 3.42×10^-3^ |
| 13q14.11 | rs4406961 | SERP2 | A/G | 0.048 | 0.085 | 0.52 | 8.83×10^-6^ | 0.47 | 1.49×10^-4^ | 0.56 | 1.74×10^-3^ |
| 14q21.1 | rs8019328 | FBXO33 | T/G | 0.342 | 0.391 | 0.76 | 3.23×10^-5^ | 0.77 | 3.10×10^-3^ | 0.75 | 7.35×10^-3^ |

^a^ MAF, minor allele frequency.

^b^ Derived from additive model with adjustment for age, gender, pack-year of smoking and the first principle component; OR_all and *P*_all were for all samples in GWAS scan; OR_nj and *P*_nj were for Nanjing samples in GWAS scan; OR_bj and *P*_bj were for Beijing samples in GWAS scan.
