# Supplementary material for: Genome-Wide Association Study Identifies a Novel Susceptibility Locus at 12q23.1 for Lung Squamous Cell Carcinoma in Han Chinese
Source: PLoS Genet. 2013 Jan 17;9(1):e1003190. doi: 10.1371/journal.pgen.1003190 (PMC3547794; doi:10.1371/journal.pgen.1003190)
Supplement: Table S4 — SNPs satisfy the selection criteria for replication but are in strong linkage disequilibrium (r2>0.8) with selected SNPs. (DOC) [file pgen.1003190.s008.doc]

**Table S4.** SNPs satisfy the selection criteria for replication but are in strong linkage disequilibrium (r^2^ > 0.8) with selected SNPs

| **Chr.** | **SNP** | **OR** ^a^ | ***P*** ^a^ | **Selected SNPs** | **r^2^** |
| --- | --- | --- | --- | --- | --- |
| 1 | rs2225904 | 1.37 | 8.15×10^-5^ | rs1445227 | 0.98 |
| 1 | rs4658549 | 0.73 | 4.38×10^-5^ | rs5009401 | 0.99 |
| 1 | rs6703812 | 0.75 | 5.34×10^-5^ | rs5009401 | 0.85 |
| 2 | rs777585 | 0.74 | 2.23×10^-5^ | rs2167566 | 0.93 |
| 2 | rs2290324 | 0.76 | 9.06×10^-5^ | rs2167566 | 0.94 |
| 2 | rs2600672 | 0.76 | 8.10×10^-5^ | rs2167566 | 0.94 |
| 2 | rs778157 | 0.76 | 7.12×10^-5^ | rs2167566 | 0.96 |
| 2 | rs778143 | 0.76 | 7.94×10^-5^ | rs2167566 | 0.92 |
| 7 | rs7787744 | 1.47 | 2.09×10^-5^ | rs10952289 | 0.99 |

^a^ OR and *P* values were derived from additive model for all samples in GWAS scan with adjustment for age, gender, pack-year of smoking and the first principle component.
