# Supplementary material for: Genome-Wide Association Study Identifies a Novel Susceptibility Locus at 12q23.1 for Lung Squamous Cell Carcinoma in Han Chinese
Source: PLoS Genet. 2013 Jan 17;9(1):e1003190. doi: 10.1371/journal.pgen.1003190 (PMC3547794; doi:10.1371/journal.pgen.1003190)
Supplement: Table S5 — Summary of associations of 14 SNPs with risk of lung SqCC in GWAS scan and replication studies. (DOC) [file pgen.1003190.s009.doc]

**Table S5.** Summary of associations of 14 SNPs with risk of lung SqCC in GWAS scan and replication studies

| **Chr.** | **SNPs** | **Study** | **Cases ^b^** | **Controls ^b^** | **MAF ^c^** | | **OR_add_** | ***P*_add_ ^d^** |
| --- | --- | --- | --- | --- | --- | --- | --- | --- |
|  | **Alleles ^a^** |  |  |  | **Cases** | **Controls** | **(95% CI) ^d^** |  |
| 1p21.2 | rs1445227 | GWAS | 29/266/538 | 81/850/2161 | 0.195 | 0.164 | 1.39(1.19-1.63) | 3.50×10^-5^ |
|  | A/G | Replication I | 28/240/547 | 84/664/1481 | 0.182 | 0.187 | 1.05(0.88-1.24) | 5.92×10^-1^ |
| 1q43 | rs5009401 | GWAS | 50/315/468 | 233/1307/1554 | 0.249 | 0.287 | 0.73(0.63-0.85) | 3.26×10^-5^ |
|  | A/G | Replication I | 57/327/415 | 159/949/1125 | 0.276 | 0.284 | 0.91(0.78-1.07) | 2.50×10^-1^ |
| 2p15 | rs2167566 | GWAS | 101/359/372 | 421/1522/1149 | 0.337 | 0.382 | 0.75(0.66-0.86) | 4.90×10^-5^ |
|  | T/G | Replication I | 109/382/321 | 347/1018/877 | 0.370 | 0.382 | 0.97(0.85-1.12) | 7.14×10^-1^ |
| 2q21.1 | rs6724110 | GWAS | 142/380/310 | 636/1544/912 | 0.399 | 0.455 | 0.78(0.69-0.89) | 9.49×10^-5^ |
|  | T/C | Replication I | 152/383/274 | 422/1115/683 | 0.425 | 0.441 | 0.93(0.82-1.07) | 3.25×10^-1^ |
| **6p22.2** | **rs16889835** | **GWAS** | **47/297/472** | **280/1259/1518** | **0.240** | **0.298** | **0.72(0.62-0.83)** | **4.49×10^-6^** |
|  | **C/T** | **Replication I** | **49/316/446** | **171/899/1155** | **0.255** | **0.279** | **0.84(0.72-0.98)** | **2.98×10^-2^** |
|  |  | **Replication II** | **148/526/631** | **443/1592/1935** | **0.315** | **0.312** | **1.00(0.90-1.10)** | **9.31×10^-1^** |
| 7q36.1 | rs10952289 | GWAS | 32/217/584 | 51/675/2367 | 0.169 | 0.126 | 1.47(1.23-1.75) | 2.23×10^-5^ |
|  | C/T | Replication I | 13/201/600 | 43/495/1692 | 0.139 | 0.130 | 1.06(0.87-1.28) | 5.90×10^-1^ |
| 9q21.11 | rs2039625 | GWAS | 41/279/508 | 219/1147/1719 | 0.218 | 0.257 | 0.71(0.61-0.83) | 1.46×10^-5^ |
|  | T/A | Replication I | 48/289/481 | 164/797/1266 | 0.235 | 0.253 | 0.96(0.82-1.11) | 5.75×10^-1^ |
| 10q11.21 | rs10900189 | GWAS | 49/292/471 | 282/1171/1560 | 0.240 | 0.288 | 0.72(0.62-0.83) | 3.45×10^-6^ |
|  | C/T | Replication I | 67/300/449 | 173/840/1210 | 0.266 | 0.267 | 1.01(0.87-1.18) | 8.50×10^-1^ |
| 11p14.1 | rs10835161 | GWAS | 141/414/278 | 709/1527/855 | 0.418 | 0.476 | 0.76(0.67-0.86) | 1.06×10^-5^ |
|  | A/C | Replication I | 178/402/236 | 444/1150/630 | 0.465 | 0.458 | 1.05(0.91-1.2) | 5.27×10^-1^ |
| **11p15.1** | **rs7112278** | **GWAS** | **36/253/544** | **206/1158/1729** | **0.195** | **0.254** | **0.71(0.62-0.83)** | **8.84×10^-6^** |
|  | **T/C** | **Replication I** | **25/275/514** | **123/782/1319** | **0.200** | **0.231** | **0.78(0.66-0.92)** | **2.95×10^-3^** |
|  |  | **Replication II** | **130/511/679** | **384/1456/2085** | **0.292** | **0.283** | **1.05(0.95-1.16)** | **3.56×10^-1^** |
| 12p13.31 | rs216905 | GWAS | 13/199/620 | 22/532/2537 | 0.135 | 0.093 | 1.52(1.24-1.85) | 4.61×10^-5^ |
|  | C/T | Replication I | 18/152/650 | 32/427/1772 | 0.115 | 0.110 | 1.05(0.85-1.29) | 6.56×10^-1^ |
| **12q23.1** | **rs12296850** | **GWAS** | **35/287/511** | **185/1204/1705** | **0.214** | **0.254** | **0.73(0.63-0.86)** | **9.30×10^-5^** |
|  | **A/G** | **Replication I** | **38/254/517** | **127/856/1241** | **0.204** | **0.250** | **0.75(0.63-0.88)** | **5.08×10^-4^** |
|  |  | **Replication II** | **86/447/832** | **289/1547/2235** | **0.227** | **0.261** | **0.82(0.74-0.91)** | **3.47×10^-4^** |
|  |  | **Combined All** | **159/988/1860** | **601/3607/5181** | **0.217** | **0.256** | **0.78(0.72-0.84)** | **1.19×10^-10^** |
| 13q14.11 | rs4406961 | GWAS | 0/79/745 | 32/460/2584 | 0.048 | 0.085 | 0.52(0.39-0.7) | 8.83×10^-6^ |
|  | A/G | Replication I | 1/121/690 | 11/335/1878 | 0.076 | 0.080 | 1.05(0.81-1.35) | 7.35×10^-1^ |
| 14q21.1 | rs8019328 | GWAS | 94/379/355 | 475/1445/1146 | 0.342 | 0.391 | 0.76(0.67-0.87) | 3.23×10^-5^ |
|  | T/G | Replication I | 120/372/311 | 337/1014/873 | 0.381 | 0.380 | 0.99(0.86-1.13) | 8.67×10^-1^ |

^a^ Major/Minor alleles;

^b^ Minor homozygote/Heterozygote/Major homozygote;

^c^ MAF, Minor allele frequency;

^d^ Derived from additive model with adjustment for age, gender, pack-year of smoking and the first principal component in GWAS, or for age, gender and pack-year of smoking in replication studies.
