# Supplementary material for: Genome-Wide Association Study Identifies a Novel Susceptibility Locus at 12q23.1 for Lung Squamous Cell Carcinoma in Han Chinese
Source: PLoS Genet. 2013 Jan 17;9(1):e1003190. doi: 10.1371/journal.pgen.1003190 (PMC3547794; doi:10.1371/journal.pgen.1003190)
Supplement: Table S6 — SNPs at 12q13.1 associated with risk of lung SqCC at P<1.0×10^−4^. (DOC) [file pgen.1003190.s010.doc]

**Table S6.** SNPs at 12q13.1 associated with risk of lung SqCC at *P* < 1.0×10^-4^

| **SNP** | **Position** | **Alleles** ^a^ | **MAF** ^b^ | **OR_add_** ^c^ | ***P*_add_** ^c^ | **Status** |
| --- | --- | --- | --- | --- | --- | --- |
| rs12296850 | 99344216 | A/G | 0.214/0.254 | 0.73 | 9.30×10^-5^ | Genotyped |
| rs17030141 | 99326955 | A/G | 0.213/0.246 | 0.83 | 6.46×10^-5^ | Imputed |
| rs11568535 | 99330846 | G/T | 0.213/0.245 | 0.83 | 7.43×10^-5^ | Imputed |

^a^ Major/Minor alleles;

^b^ Minor allele frequency for cases/controls;

^c^ Derived from additive model with adjustment for age, gender, pack-year of smoking and the first principal component.
