# Supplementary material for: Genome-Wide Association Study Identifies a Novel Susceptibility Locus at 12q23.1 for Lung Squamous Cell Carcinoma in Han Chinese
Source: PLoS Genet. 2013 Jan 17;9(1):e1003190. doi: 10.1371/journal.pgen.1003190 (PMC3547794; doi:10.1371/journal.pgen.1003190)
Supplement: Table S7 — Stratification analysis on association of rs12296850 at 12q13.1 and lung SqCC risk. (DOC) [file pgen.1003190.s011.doc]

**Table S7.** Stratification analysis on association of rs12296850 at 12q13.1 and lung SqCC risk

| **Variables** | **GG/AG/AA genotypes** | | **OR (95% CI) ^a^** | ***P* ^a^** | ***P* ^b^** |
| --- | --- | --- | --- | --- | --- |
|  | **Case** | **Control** |  |  |  |
| **Age** |  |  |  |  | 0.737 |
| **≤60** | 89/463/916 | 328/1887/2768 | 0.79(0.71-0.88) | 1.08×10^-5^ |  |
| **>60** | 70/525/944 | 273/1720/2413 | 0.77(0.69-0.85) | 1.47×10^-6^ |  |
| **Gender** |  |  |  |  | 0.457 |
| **Male** | 139/875/1593 | 420/2391/3393 | 0.79(0.73-0.86) | 1.90×10^-8^ |  |
| **Female** | 20/113/267 | 181/1216/1788 | 0.73(0.60-0.88) | 9.25×10^-4^ |  |
| **Smoking status** |  |  |  |  | 0.801 |
| **Never** | 38/182/379 | 333/2145/3087 | 0.81(0.70-0.94) | 3.74×10^-3^ |  |
| **0-25** | 31/226/417 | 138/757/1149 | 0.80(0.69-0.93) | 2.49×10^-3^ |  |
| **>25** | 90/580/1064 | 130/705/945 | 0.76(0.67-0.88) | 3.50×10^-7^ |  |

^a^ Derived from additive model with adjustment for age, gender and pack-year of smoking where is appropriate.

^b^ Test for heterogeneity.
